# Editorial: Progress and Prospects on Skin Imaging Technology, Teledermatology and Artificial Intelligence in Dermatology

**DOI:** 10.3389/fmed.2021.757538

**Published:** 2021-11-12

**Authors:** Chengxu Li, Je-Ho Mun, Paola Pasquali, Hang Li, H. Peter Soyer, Yong Cui

**Affiliations:** ^1^Department of Dermatology, China-Japan Friendship Hospital, Beijing, China; ^2^Department of Dermatology, Seoul National University College of Medicine, Seoul, South Korea; ^3^Department of Dermatology, Pius Hospital de Valls, Tarragona, Spain; ^4^Department of Dermatology, Peking University First Hospital, Beijing, China; ^5^The University of Queensland Diamantina Institute, The University of Queensland, Dermatology Research Centre, Brisbane, QLD, Australia

**Keywords:** skin imaging technology, dermoscopy, reflectance confocal microscopy, teledermatology, artificial intelligence in dermatology, big data in dermatology

## Introduction

Dermatology is a clinical discipline based on intuitive features. With the emergence of digital technology, remote transmission, and internet technologies, dermatology has become a very well-suited discipline to integrate these technologies and apply them in clinical practice due to its characteristics. At present, the development of skin imaging technologies [dermoscopy, reflectance confocal microscopy (RCM), optical coherence tomography (OCT), etc.], teledermatology, and artificial intelligence (AI) has profoundly and comprehensively changed the nature, service model, and public recognition of dermatology (1). Skin imaging, as an important technical system in modern dermatology, continues to gain the attention of researchers and the wider community alike. In this spirit, we have proposed a Research Topic titled “Progress and Prospects on Skin Imaging Technology, Teledermatology and Artificial Intelligence in Dermatology” and are very pleased indeed that nine manuscripts on the various aspects of skin imaging technology, five manuscripts on teledermatology, and seven manuscripts on AI in dermatology have been published under the banner of this specific Research Topic. In the current article, we aimed to introduce 21 articles published in this specific Research Topic, so that readers can refer to the articles more pertinently.

## Updates On Skin Imaging Technology

Skin imaging, as a non-invasive imaging technology, is widely used in disease diagnosis (2), treatment follow-up (3), and surgical boundary determination (4). The combined use of different skin imaging equipment and the combination of skin imaging and AI have further improved the diagnostic accuracy of skin imaging and broadened its application range (5, 6).

In terms of research on dermoscopy, Wang et al. identified new dermatoscopic features through a morphological study of 39 patients with acquired perforating dermatosis. Relative to previously reported dermoscopic features, the newly identified features such as the dam shape uplift at the periphery may contribute to assist clinical diagnosis. Chen et al. investigated the trichoscopic features of female pattern hair loss in Chinese Han patients, analyzed the difference between different genders and pointed out female pattern hair loss could occur in male with characteristics similar to female. Gao et al. provided a comprehensive summary of the dermoscopic features for four common cutaneous vascular anomalies, namely, infantile hemangioma, cherry angioma, angiokeratoma, and pyogenic granuloma in the Chinese Han population. They described that dermoscopic features of cherry angioma and the other common vascular tumors vary in age stages and different anatomical sites. Chanprapaph et al. used nailfold capillaroscopy technology to investigate nailfold features in patients with systemic lupus erythematosus, dermatomyositis, and systemic sclerosis, and tried to find out which features could distinguish these connective tissue diseases. In addition, the authors explored different capillaroscopic abnormalities and their association with disease activity.

In terms of RCM applications, Feng et al. summarized confocal features of lesions in 11 patients with syringoma on the vulva. The authors compared RCM features with the biopsy findings for histopathological correlation and then highlighted the potential role of RCM in the diagnosis and differential diagnosis of vulva syringoma. Liu et al. studied persistent hypopigmented patches suspicious of early-stage vitiligo or hypopigmented mycosis fungoides with RCM. All the imaged lesions were biopsied and analyzed by histopathology. Their findings indicated that RCM could be a useful tool to screen for hypopigmented mycosis fungoides in hypopigmented lesions underlining. The combined use of dermoscopy and RCM may lead to new diagnostic indications. Wang et al. described dermoscopy and confocal characteristics of two patients with primary cutaneous amyloidosis in two different histopathological subtypes. Although this disease usually is suspected clinically, for cases with an atypical presentation, dermoscopy combined with RCM can be used as a reference for clinicians.

In the application of high-frequency ultrasound, Li et al. conducted a retrospective study to investigate the ultrasonographic features of pilomatricoma and determined the associations of these characteristics with clinical features in different histopathological subtypes. Ultra-high-frequency was proved to be a useful tool to diagnose and stage this disease before surgery. Due to the unique structure and function of the nail unit (NU), it is particularly important to develop useful non-invasive inspection methods. Lipska et al. described that high-frequency ultrasonography was promising in the visualization of the NU structure although in some cases the image quality was insufficient. In addition to ultrasonographic features, the authors measured transonychial water loss by evaporimetry.

All these papers utilizing skin imaging technologies such as dermoscopy, RCM and high-frequency ultrasound, have in common that still numerous inflammatory and neoplastic skin conditions can be studied with imaging technologies leading to a more objective characterization of the morphologic features of the many faces of skin pathologies.

## Updates On Teledermatology

Teledermatology is an effective approach to decrease the differences in healthcare quality among regional and metropolitan areas and provide an overall improvement. Teledermatology is enabled by remote transmission technology and internet technology and is gradually changing the way of healthcare access. Endeavors to provide remote consultation and care through telemedicine platforms have started in the last years in several countries. Live interactive technology has enabled remote controlled access to RCM devices and guided clinicians toward diagnosis, breaking through the limitations of existing store-and-forward teledermatology (7). Especially during the COVID-19 pandemic, teledermatology played a very important role and its development was significantly promoted (8).

The following few articles represent a small snapshot on the progress in this field. Edwards et al. summarized the strategy for teledermatology in the COVID-19 period in the dermatology department at Princess Alexandra Hospital, in Brisbane, Australia. A system of telephone consultations and store-and-forward imaging were used to deal with the situation of the epidemic. The efficacy of this system provides experience to develop future telemedicine systems. Giavina-Bianchi et al. compared the consistency of diagnosis made by teledermatologists and in-person dermatologists and presented a high accuracy in the diagnosis of the 20 most frequent inflammatory dermatoses using teledermatology. Besides, their study discovered moderate consistency between teledermatologists, histopathological reports, and in-person dermatologists' diagnosis in skin neoplasms, which reassures the potential of store-and-forward teledermatology being an option for patient care with skin cancer. Polańska et al. present the current applications of 20-MHz ultrasonography in dermatology, including skin tumors and chronic skin diseases. Moreover, the authors analyzed the possibilities for high-frequency ultrasonography being used as a tool in teledermatology, especially in the diagnosis and monitoring of patients with chronic skin diseases, such as psoriasis, atopic dermatitis, vitiligo, or leg ulcers. The convergence of teledermatology with software has driven the management of chronic skin diseases. Stark et al. introduced how IMPROVE 1.0, an android app monitoring psoriasis patients by questionnaires, helped doctors and patients to assess and study disease activity. And it demonstrated smartphone apps' value in disease management and doctor-patient communication.

## Updates On Artificial Intelligence In Dermatology

Artificial intelligence is strengthening dermatologists (9). Especially for atypical, challenging, or doubtful cases, AI algorithms could be a useful tool for the dermatologists (10). Based on big data made of high-quality skin images (including skin photos, dermoscopy images, skin confocal images, skin pathology images), AI assists dermatologists in making clinical decisions in diagnosis, evaluation, and treatment. Progress has been achieved through new efforts in the development and application of AI support in dermatology, such as the artificially intelligent diagnosis algorithm for skin cancer by Stanford researchers based on the US data (11), the International Skin Imaging Collaboration (ISIC) with its annual challenges and China's Chinese Skin Image Database (12). Diagnosis of skin diseases is often challenging, and computer-aided diagnostic tools are urgently needed to underpin decision making. Thomsen et al. used non-standardized images to train a VGG-16 model to identify acne from rosacea and cutaneous T-cell lymphoma from eczema. The performance rates reported were equal or superior to those reported for general practitioners with dermatological training, indicating that computer-aided diagnostic models based on the convolutional neural network have the potential in diagnosing multiple-lesion skin diseases. Zhu et al. applied Google's EfficientNet-b4 with pre-trained weights on ImageNet as the backbone of the CNN architecture and trained it using labeled dermoscopic images of 14 common skin diseases. The test results show that the proposed framework has high classification performance, which is superior to previously reported methods. Also, the performance of this CNN model was comparable to 280 board-certified dermatologists in an eight-class diagnostic task, with higher sensitivity in all of the included diseases. Onychomycosis is a common fungal nail infection, and accurate diagnosis is important. Lim et al. reviewed the main characteristics of traditional diagnostic tools for onychomycosis and the newly developed technologies, including dermoscopy, RCM, molecular assays, and AI.

The role of the Digital Imaging and Communications in Medicine (DICOM) standard in AI for skin disease was reviewed. Although DICOM is common in some medical image-producing specialties, the application of DICOM in dermatology imaging has been limited. Caffery et al. discussed the role of DICOM in AI from the perspective of skin imaging and reviewed the current status of AI-specific content in the DICOM standard. DICOM can improve AI workflows by encoding derived objects (e.g., secondary images, visual explainability maps, AI algorithm output) and the efficient curation of multi-institutional datasets for machine learning training, testing, and validation.

Chinese dermatologists' attitudes toward AI have been investigated before, and the vast majority of interviewees believe that the role of AI is to assist dermatologists in diagnosis and treatment (13). Polesie et al. conducted a survey on AI in dermatopathology and reported that 848 dermatopathologists worldwide expected AI for handling narrowly specified tasks, such as automated detection of mitoses and tumor margins as well as for immunostaining evaluation, rather than a global automated suggestion of diagnoses. To understand how to better help dermatologists CNN's inner workings, Dalmau et al. developed an online hands-on pedagogical activity as a tool for beginners to learn about CNNs. Such an attempt is of great significance for clinicians including dermatologists accessing computer science and we encourage and appreciate further solutions in this context.

## Conclusion

In sum, this special Research Topic of skin imaging technology, teledermatology, and AI in dermatology collects the latest endeavors, progresses, experiences, and challenges in these fields and shows the successful integration of modern information technology into dermatology. Through these studies and investigations, the expectations and positioning of AI in dermatology have been clarified, the applicability of skin imaging techniques has been expanded, and the changes and development of teledermatology in different contexts have been driven. In our estimation, in the future these three essential elements could offer powerful support for patients and doctors alike and even become a mainstream medical model.

## Author Contributions

All authors listed have made a substantial, direct and intellectual contribution to the work, and approved it for publication.

## Funding

This work has been supported by the Beijing Municipal Science and Technology Commission Medicine Collaborative Science and Technology Innovation Research Project (No. Z191100007719001). HS holds an NHMRC MRFF Next Generation Clinical Researchers Program Practitioner Fellowship (APP1137127).

## Conflict of Interest

HS is a shareholder of MoleMap NZ Limited and e-derm consult GmbH, and undertakes regular teledermatological reporting for both companies. HS is a Medical Consultant for Canfield Scientific Inc., MoleMap Australia Pty. Ltd., Revenio Research Oy, and a Medical Advisor for First Derm. The remaining authors declare that the research was conducted in the absence of any commercial or financial relationships that could be construed as a potential conflict of interest.

## Publisher's Note

All claims expressed in this article are solely those of the authors and do not necessarily represent those of their affiliated organizations, or those of the publisher, the editors and the reviewers. Any product that may be evaluated in this article, or claim that may be made by its manufacturer, is not guaranteed or endorsed by the publisher.
